# Multi-Criteria Decision-Making Framework for Sustainable Innovation Management in the Mexican Medical Device Manufacturing Industry: An Exploratory and Interdisciplinary Analysis

**DOI:** 10.3390/jmahp14030041

**Published:** 2026-07-27

**Authors:** José Cozain-Hernández, Josué Aarón López-Leyva, Miguel Angel Ponce-Camacho, Víctor Manuel Ramos-García

**Affiliations:** 1 Colegio de Ingeniería, CETYS Universidad, Tijuana 22210, Mexico; jose.cozain@gmail.com; 2 Colegio de Ingeniería, CETYS Universidad, Ensenada 22860, Mexico; 3 Colegio de Ingeniería, CETYS Universidad, Mexicali 21259, Mexico; miguel.ponce@cetys.mx; 4 Departamento de Física, Matemáticas e Ingeniería, Universidad de Sonora, Navojoa 85880, Mexico; victor.ramosgarcia@unison.mx

**Keywords:** innovation management, medical device industry, sustainable, multi-criteria

## Abstract

The medical device manufacturing industry in Mexico faces a critical risk of losing competitiveness and sustainability due to its concentration on low-value-added manufacturing activities and limited integration into advanced stages of the value chain, such as R&D. This research addresses the lack of validated quantitative methodologies to identify the critical factors that promote sectoral sustainability in the national context. Through a literature review and the analysis of MCDM, a taxonomy of the problem was developed that integrates dimensions of governance, technological innovation, and human capital. The findings emphasize the need to transition toward circular economy and additive manufacturing models, supported by hybrid algorithms such as AHP, TOPSIS, and DEMATEL to mitigate uncertainty in strategic decision making. As a main result, an innovation management flow aligned with international standards and several maturity levels (TRLs, MRLs, CRLs, and PRLs) is proposed, providing a structured roadmap to scale the Mexican industry toward more-sophisticated global segments.

## 1. Introduction

The medical device sector is one of the most important industries in the world. In fact, it is experiencing steady growth, with projected global sales of almost $800 billion USD by 2030, which represents an annual increase of 5% [1]. In addition, it is estimated to employ around 1.13 million people worldwide and encompasses products ranging from simple items such as gauze and adhesive bandages to complex devices that perform vital bodily functions [2]. In particular, Mexico is a significant contributor to this manufacturing sector, with exports totaling $13.964 billion USD in 2024, accounting for around 1.8% of the country’s total workforce [3].

In order to understand Mexico’s position within the global framework, it is important to identify its role in the supply and value chains of the medical device industry. In general, the supply chain involves the acquisition, transportation, and receipt of all the raw materials and inputs necessary for manufacturing goods. In the case of Mexico’s medical device industry, part of its success is due to its proximity to the United States of America, with which it maintains close economic relations, facilitating the import of production resources. This also facilitates the export of finished and semi-finished products.

By contrast, an analysis of Mexico’s position within the value chain reveals that its contribution is focused on the production (i.e., manufacturing or assembly) of medical devices, with less development in other stages of the product life cycle, such as R&D and supply chain development [4]. This represents a competitive disadvantage compared with countries such as Costa Rica, which have invested in diversifying their capabilities over the last two decades, for example, by creating a Master’s Program in Innovation in Medical Devices and committing to moving up the value chain in R&D activities through incentives and government policies [5]. Consequently, there is a risk that Mexico will lose market share and become unsustainable in the long term.

Considering this, there exists research, such as that by Gereffi and Hamrick (2026) [4], that addresses this issue by evaluating the industry’s needs in the region from a political and economic perspective. These authors argue that the sector’s competitiveness depends on public policies that focus on supplier development, technical skill development, and institutional coordination. However, no quantitative or objective evaluation is presented to substantiate this affirmation. In fact, their significance is based on opinions or qualitative analyses without a documented foundation. In particular, Salazar (2022) [5] demonstrates the growth associated with public policies and academic development in a case study of the medical device industry in Costa Rica; however, this study also fails to provide an evaluation proving that these factors are the most relevant for determining a nation’s success in entering this industry. In addition, Montesinos et al. (2024) [6] address the problem of sustainability by examining 41 different studies, ultimately concluding that economic, environmental, and social aspects significantly affect this goal. However, the study highlights that other factors may exist.

Without a validated methodology to identify and act on the most significant factors for promoting the sustainability of Mexico’s medical device industry, there is a risk of significant market share loss relative to other regions with greater participation across the rest of the value chain. This would risk the employment of 1.8% of the Mexican workforce, as well as this sector’s contribution to the nation’s economic activity.

To understand the current state of the industry and the factors that contribute to its sustainability, this research begins with a review of the existing literature on the elements that constitute this sector, including the supply chain, logistics, governance, and regulations. In addition, a review was conducted of previous efforts to apply the multi-criteria decision analysis tools that could be used for the management of innovation in that industrial sector. This review identified important information that could contribute to the research, as well as opportunities that had not yet been explored and could be addressed by this study. Thus, based on this comprehensive view of the industry, the study subsequently presents research and innovation proposals that aim to generate new information or actionable knowledge to support Mexico’s development in this sector.

Therefore, the purpose of this research is to review and analyze hybrid methods for decision making for innovation management to solve the open challenges in the medical device manufacturing industry in México. Thus, the paper is a narrative review.

## 2. Problem Taxonomy

**Figure 1:**
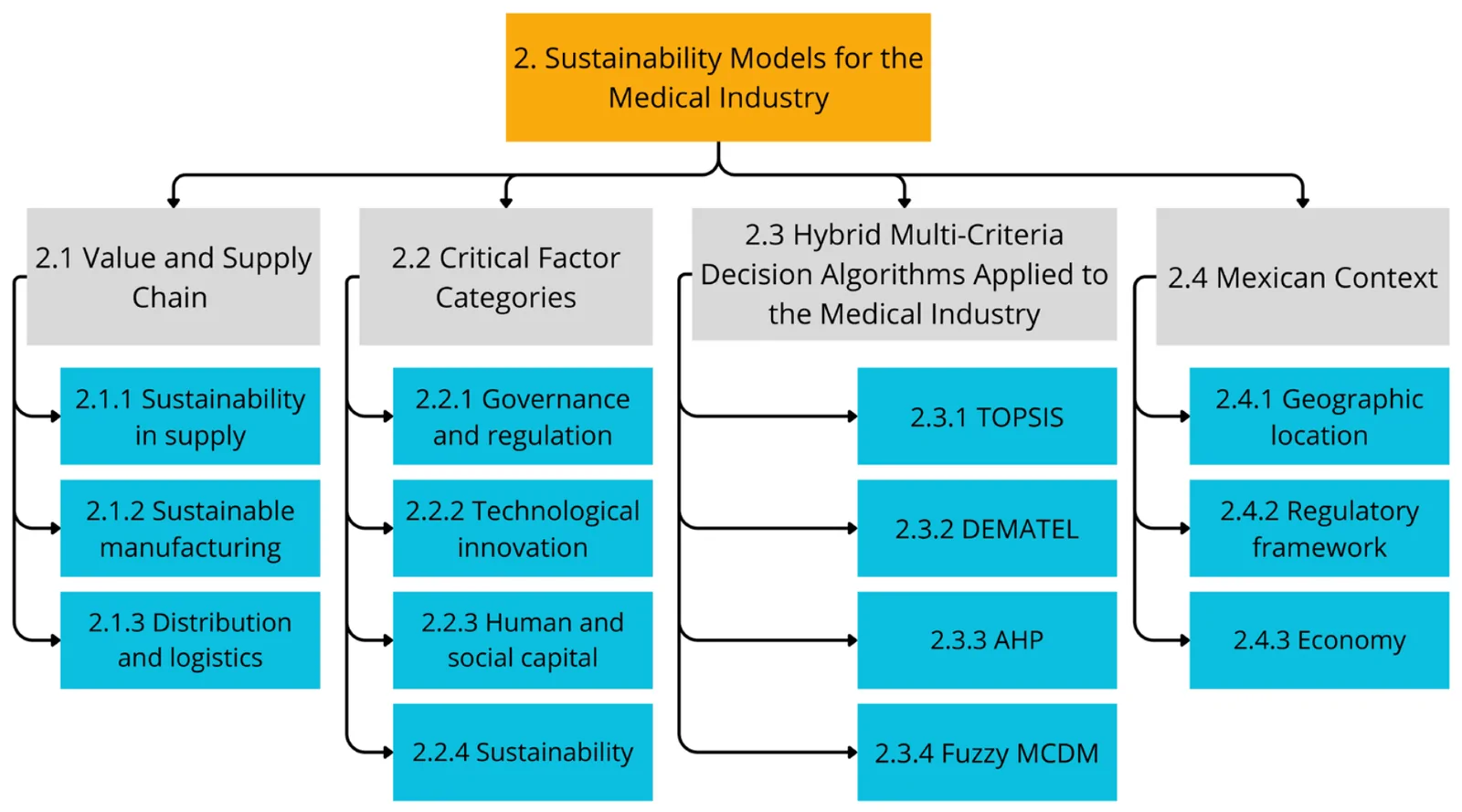
Problem taxonomy.

With the purpose of identifying and addressing in a structured manner the topics necessary to carry out comprehensive research, the following taxonomy was established (see Figure 1). The concept of “Sustainability Models for the Medical Industry” is the guiding principle at the first level. The second-level categories emerge from this concept, which are Value and Supply Chain, Critical Factor Categories, Hybrid Multi-Criteria Decision Algorithms Applied to the Medical Industry, and finally, the Mexican Context. The first two research lines address the internal and external factors that directly affect the value chain and the medical device industry in general. The category of hybrid algorithms includes hybrid multi-criteria decision algorithms, such as Technique for Order of Preference by Similarity to Ideal Solution (TOPSIS), Decision-Making Trial and Evaluation Laboratory (DEMATEL), Analytic Hierarchy Process (AHP), and Fuzzy Multi-Criteria Decision Making (MCDM). These are used to determine objectively and on a scientific basis which factors are key. The fourth research line, the Mexican Context, identifies factors that influence sustainability specific to the region.

The structure shown in Figure 1 was developed considering the authors’ experience in the medical device manufacturing sector within the Mexican context. Furthermore, the development of each category was based solely on documents no older than five years. In this regard, the key search terms included: “medical device” AND “Mexico” AND (“market access” OR “technology transfer” OR “health policy”). In addition, manual searches were conducted in the reference lists of key articles.

## 3. Literature Review

Reviewing the existing literature on the various aspects of the medical device industry provides a comprehensive overview of the research problem. This review identifies which factors have been evaluated to support the industry and which factors have not been addressed sufficiently or at all and therefore warrant further research. In terms of value and supply chains, particularly with regard to supply sustainability, a viable strategy for increasing the sustainability and availability of medical devices has been identified: combining Original Equipment Manufacturing (OEM) with remanufacturing by certified Contract Manufacturers (CMs). This approach increases the quantity of mechanisms and mitigates the impact of supply chain disruptions [7]. For its part, selecting suppliers in a way that contributes to supply sustainability is itself a challenge. Potential candidates must be evaluated based on more than just economic factors; indicators of sustainability, Industry 4.0 maturity, and *LeAgile* (a combination of Lean and Agile concepts) must also be considered to prioritize and select suppliers [8]. The impact of human capital (i.e., the people and their knowledge, experience, and skills that make up a company) has also been found to have a significant indirect impact on large companies through their cost and quality strategies [9]. Leong (2025) [10], on the other hand, proposes a circular economy system through additive manufacturing with a focus on sustainability. This system contributes to the industry’s sustainability by separating value creation from the use of virgin natural resources. This study’s key contributions include proposing additive manufacturing close to sites of use (e.g., hospitals) and creating design patterns for circular manufacturing. Regarding technological innovation, the use of emerging technologies such as the Internet of Things (IoT) and blockchain for obtaining and distributing relevant information among all participants in the supply chain allows for a solid and reliable integration between manufacturers, suppliers, healthcare organizations, end users, and government entities, thus strengthening the chain and making it more resilient [11]. Also, due to the large number of alternatives and the complexity of the supply chain optimization and sustainability problem in both the medical industry and the production industry in general, various efforts have made use of hybrid MCDM, which is a part of operations research, combining mathematical and computational tools to perform a subjective evaluation of the performance criteria set by decision-makers [12]. In general, there are numerous MCDM methods that have been used to evaluate elements to optimize in the medical industry’s value chain. For instance, a supply chain sustainability analysis used a combination of TOPSIS, Complex Proportional Assessment (COPRAS), Weighted Aggregate Sum of Products Assessment (WASPAS), and Combined Compromise Solution (CoCoSo). This analysis compared different contributors and concluded that collaboration and communication, as well as blockchain technology, have a significant impact on sustainability [13]. Another important component of these studies is fuzzy logic, which is part of MCDM. In particular, fuzzy logic is associated with uncertainty and is an artificial intelligence technique that uses linguistic terms to perform reasoning and facilitate the analysis and interpretation of imprecise information [14]. Also, Agrawal et al. (2025) [15] performed a fuzzy DEMATEL analysis and subsequent linguistic analysis based on expert opinions to determine the factors, interrelationships, and priorities that influence supply chains for implementing circular logistics, finding “long-term strategic planning” as the main factor. In the Mexican context specifically, Baja California is the leading contributor to medical device production nationwide, employing around 100,000 people in 128 companies [4]. One of the main incentives for foreign direct investment is the manufacturing model that originated in 2007 when various export incentive programs were consolidated into the IMMEX program [16]. Also, the government agency that regulates medical devices is COFEPRIS. Despite the above, the United States of America has reported delays of up to 24 months in sanitary registration and import permit applications and considers these delays to be the main barrier to entering the Mexican market [17]. Table 1 shows a comparative analysis of the most relevant research.

## 4. Identifying Gaps and Open Challenges

The literature review presents relevant information that contributes to the development of potential innovation projects and highlights significant gaps that must be explored to determine the factors that promote sustainability in Mexico’s medical device industry with a high degree of certainty.

The first open challenge identified is adapting research findings to the Mexican context. A review of previous research efforts revealed no articles addressing Mexico’s specific situation quantitatively or objectively. Therefore, a deeper examination of the region’s unique challenges is necessary to develop an adapted roadmap for the nation. This focused research should identify the factors that influence the Mexican context specifically and whether they differ from those applicable in the global context.

Regarding the methodology employed, there is an open challenge to determine the most appropriate MCDM method. In general, previous studies have employed techniques such as DEMATEL, AHP, TOPSIS, and hybrid methods. However, no evaluation confirms that these methods are the most suitable. In addition, some studies suggest exploring other methodologies. For example, Agrawal et al. (2025) [15] suggest using the Full Consistency Method (FUCOM) methodology for further analysis. Therefore, there is a need to evaluate different MCDM methods (including fuzzy variants) and use that information to structure a more reliable analysis.

Another significant challenge that was consistently identified in the literature review is the validation of expert opinions. Although these opinions form the basis of multi-criteria assessment tools, they may be biased depending on factors such as region and area ofexpertise. This makes it difficult to confirm that the collected opinions are representative of the system being evaluated.

## 5. Innovation Management to Solve the Open Challenges

Having identified the current challenges and gaps, it is clear that a specialized innovation management workflow is needed for the medical device sector. This model must balance creative freedom with a rigorous regulatory framework based on analytical processes and intelligent decision making. To ensure global compliance, this process must be aligned with internationally recognized standards, such as ISO 13485:2016 for quality management systems in the medical device industry, and the regulations of reference agencies such as the Food and Drug Administration (FDA) in the United States of America and the European Medicines Agency (EMA) [18].

### 5.1. Opportunity Identification and Technological Monitoring

In particular, innovation is not just about creative ideas; it is about systematically identifying unmet clinical needs. This methodological approach requires the implementation of a proactive technology surveillance, which involves more than just passive monitoring. It also incorporates prospective patent analysis, tracking the evolution of international regulatory frameworks and disruptive technologies such as artificial intelligence applied to diagnostic imaging or the development of advanced bioactive biomaterials. Thus, converging this data enables trends to be anticipated and technical risks to be mitigated before the design phase. In addition, it is essential to incorporate the “voice of the customer” in healthcare settings through an ethnographic approach. For instance, direct observation in operating rooms and intensive care units is essential for identifying ergonomic deficiencies and usability faults in medical devices and instruments that are often overlooked by traditional survey methods. Thus, the identification of opportunities is consolidated as a hybrid process that balances the rigor of big data analysis with empirical clinical reality, ensuring that the flow of innovation management is geared towards solving critical health problems. This comprehensive approach ensures the clinical relevance of the proposed development, optimizes commercial viability, and promotes proactive regulatory compliance in a sector characterized by high complexity and technical demands.

### 5.2. Front End of Innovation

In general, the front end of the innovation stage is a critical phase in which creativity must converge with methodological rigor to ensure successful clinical translation. The transition from an abstract concept to a viable medical device requires the implementation of a highly structured innovation pipeline, designed to mitigate risks in the early stages where uncertainty is at its peak. As the healthcare environment is subject to rigorous regulatory oversight, not all proposals are robust enough to reach commercial maturity, so the filtering process must be multidimensional. Thus, for an initiative to advance to the formal development phases, it must pass a comprehensive set of evaluation criteria. Firstly, technical feasibility must demonstrate that the solution is reproducible and safe. Simultaneously, a robust reimbursement model must be validated to ensure that healthcare systems and insurers recognize the clinical and economic value of the proposal. Lastly, a Freedom to Operate analysis is crucial. This study verifies that the design does not infringe third-party patents, thereby avoiding legal barriers that could compromise investment and product deployment in the global market. This strategic filtering process optimizes resource allocation and ensures that only innovations with genuine potential for clinical impact and commercial viability proceed through the development lifecycle.

### 5.3. Design Controls

In the field of medical device engineering, Design and Development under Control phase is crucial for ensuring that a project is technically viable and economically competitive. This stage is important because it prevents planning errors that, if not detected early, would lead to costly redesigns. In addition, this process involves translating user needs and clinical requirements into design inputs and transforming them into quantifiable and verifiable technical specifications. This workflow operates in conjunction with risk management under the ISO 14971:2019 standard, integrating simultaneously and bidirectionally [19]. This convergence enables patient safety to be considered as a design parameter from the earliest stages of the product lifecycle. The systematic application of this approach ensures traceability between design inputs, outputs, and verification and validation processes. This enables any potential failure modes to be identified and mitigated proactively. Therefore, the rigorous implementation of these controls ensures compliance with international quality standards and establishes a robust development framework focused on operational excellence and the device’s functional integrity in a clinical setting.

### 5.4. Rapid Prototyping and Verification

In the contemporary biomedical engineering ecosystem, as in other industrial sectors, time-to-market optimization depends on a reduction in learning cycles through agile iteration methodologies. The aforementioned is related to rapid prototyping, driven by additive manufacturing (3D printing) and computer-aided design, which enables early, resource-efficient conceptual validation. In addition, integrating high-fidelity computational simulations, such as finite element analysis and computational fluid dynamics, allows for predicting the mechanical and functional behavior of the device before incurring the high costs associated with complex tooling or final injection molds. However, agile prototyping must coexist with a rigorous verification phase to guarantee the integrity of the final product. This verification process includes controlled laboratory testing and physical stress testing. In particular, its objective is to definitively confirm that the device has been manufactured according to predefined technical specifications and meets the safety and efficacy standards required by regulatory bodies. In short, the combination of virtual experimentation, accelerated physical prototyping, and rigorous quality control protocols allows organizations to optimize the use of critical resources, proactively mitigate technical risks, and establish a sustainable competitive advantage in a dynamic and highly regulated market.

### 5.5. Clinical Validation and Regulatory Affairs

In general, the transition from a functional prototype to a marketable product requires a strategic balance of scientific rigor and regulatory compliance. Clinical validation is, therefore, not merely a technical verification phase, but the fundamental pillar that guarantees the safety and efficacy of the device by generating empirical evidence of its performance in real-world settings. To ensure a successful transition, it is essential to develop a proactive regulatory strategy that identifies the most efficient approval pathway based on the device’s risk classification and intended use. For devices in low- or moderate-complexity contexts, where substantial equivalence to an existing device can be demonstrated, there are more-agile processes for marketing approval. However, this regulatory agility must be supported by robust clinical trial protocols that meet the requirements of both ISO 14155:2026 and the European Medical Device Regulation [20]. In fact, integrating these regulatory issues into the design cycle from an early stage helps to mitigate technical and financial risks, ensuring that technology implementation is innovative and fully compliant with international public health standards and current quality management systems.

### 5.6. Production and Scaling

The transition to mass production and industrial scaling is a critical milestone in the medical device development lifecycle. At this stage, design integrity must be translated into efficient and reproducible operation. To achieve optimal competitiveness in terms of cost and quality, the transition from prototyping to manufacturing must be executed with impeccable technical rigor. This process is based on the strategic integration of Lean Manufacturing principles, which optimize assembly lines by systematically identifying and eliminating waste, minimizing variability, and maximizing added value at each stage of the production process. This operational flow not only seeks logistical efficiency but is also strengthened by a robust process validation methodology related to Installation Qualification, Operational Qualification, and Performance Qualification. Such validation is essential to ensure that each manufactured unit is technically identical and consistently meets the strictest safety and clinical efficacy standards required by international organizations. By harmonizing the agility of lean manufacturing methods with proactive, risk-based quality control, organizations ensure overall value chain efficiency and facilitate a reduced time-to-market without compromising regulatory compliance. Ultimately, successful scale-up ensures that the performance demonstrated under controlled laboratory conditions remains unchanged during large-scale production.

### 5.7. Launch and Post-Marketing Monitoring

Finally, the culmination of the launch phase does not represent the end of the innovation process, but rather the beginning of a critical stage of monitoring and technical optimization. In this way, Post-Marketing Surveillance is established as a dynamic component of quality management, where the systematic collection of data on clinical performance and safety under real-world use conditions is imperative. This approach transcends basic regulatory compliance to become a strategic feedback loop; by integrating usability metrics and adverse events into the product lifecycle, organizations can transfer knowledge to the design and development phase of new iterations. This flow of information allows the transition to a “Generation 2.0” of the product or medical device to be a process grounded in empirical evidence, refining therapeutic efficacy and mitigating risks previously unidentified in controlled clinical trials. Consequently, the implementation of a robust post-marketing surveillance system not only guarantees patient safety in accordance with ISO 13485:2016 [18] standards and FDA or EMA regulations but also consolidates a sustainable competitive advantage. Thus, by anticipating market needs and proactively correcting operational gaps, the company secures its position at the forefront of technology, transforming post-marketing data into the main driver of continuous improvement and disruptive innovation in the healthcare ecosystem.

### 5.8. MCDM and Maturity Levels in the Value Chain

**Figure 2:**
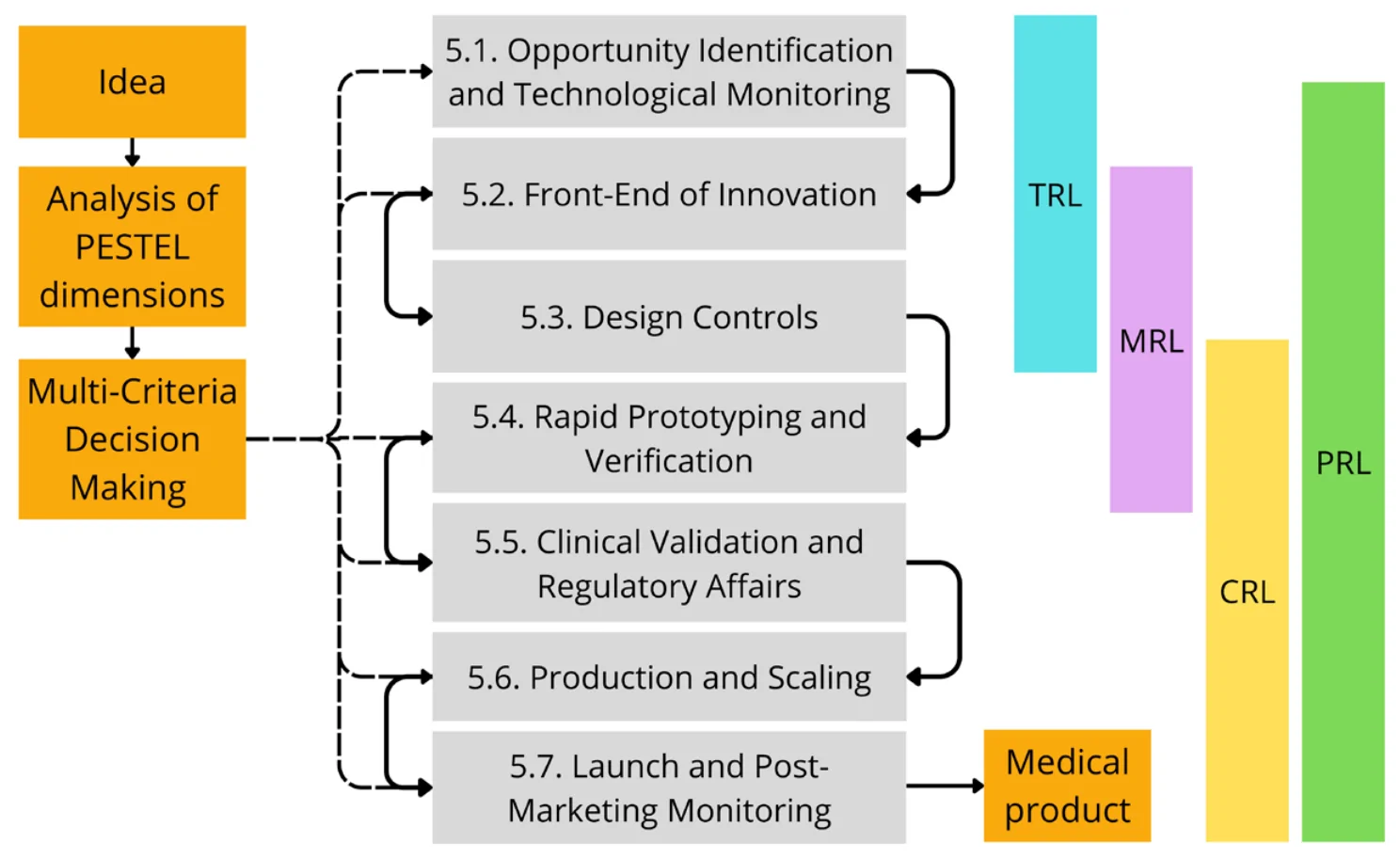
Relationship of the use of MCDM methods to impact the value chain of the medical device and product manufacturing industry.

Figure 2 clarifies the comprehensive system of relationships among all the aforementioned elements. The process begins with an innovative idea relating to a medical product or device. It is important to note that innovation can refer to either the creation of a completely new device or a significant improvement to an existing one. Next, a PESTEL analysis is conducted to identify and evaluate external factors that could impact the performance, viability, or success of an innovation in the medical manufacturing sector [21,22]. After the qualitative PESTEL (Political, Economic, Social, Technological, Environmental, and Legal) analysis, MCDM methods are applied to determine critical factors and inform customized decisions for each element of the value chain (represented by dashed lines). The various innovations are managed at different stages of the value chain, and their direct relationships are represented by solid lines. A key point to note is how the various maturity levels, such as Technology Readiness Levels (TRLs), Manufacturing Readiness Levels (MRLs), Customer Readiness Levels (CRLs), and Policy Readiness Levels (PRLs), interact with the value chain flow, which is affected by the results of the MCDM methods. Specifically, TRLs consider the evolution of technical knowledge from basic research to system validation in real-world environments. MRLs, on the other hand, measure the capacity to produce technology in a scalable and controlled manner. Then, CRLs determine market acceptance and alignment with end-user needs, while PRLs ensure compliance with current legal, ethical, and regulatory frameworks. Together, these indicators ensure a robust transition from prototype to successful commercialization [23].

## 6. Administrative Implications

Considering the above, there are important administrative implications for the long-term competitiveness of the medical device industry in Mexico that can also be considered in other locations around the world. As previously mentioned, the industry must transition from low-value manufacturing to higher stages of the value chain, such as R&D. From a management perspective, this requires organizations to adopt structured processes that align with international standards, such as ISO 13485:2016 [18], to ensure quality and global regulatory compliance. For a plant manager, in particular, this framework provides a technical roadmap for optimizing the allocation of limited resources using hybrid MCDM algorithms, such as AHP, TOPSIS, and DEMATEL. These tools enable objective evaluation of critical success factors, eliminating subjectivity in supplier selection and investment prioritization in disruptive technologies, such as additive manufacturing and the IoT, among other administrative applications. Moreover, in a VUCA (Volatility, Uncertainty, Complexity, and Ambiguity) environment, integrating fuzzy logic helps one reason with imprecise information and mitigate strategic risks before the design phase [24,25]. Similarly, the research emphasizes the importance of fostering institutional coordination and developing technical skills for policymakers to avoid losing market share to regional competitors. Additionally, implementing TRLs, MRLs, CRLs, and PRLs establishes a control system that guarantees innovations are technically feasible and commercially viable prior to industrial scaling. Thus, the proposed framework transforms innovation management from an abstract, creative process into a rigorous, analytical discipline, strengthening supply chain resilience and promoting a sustainable, circular economy.

## 7. Conclusions

The comprehensive analysis of the medical device industry presented in this paper reveals that, although Mexico has established itself as a key player in global manufacturing, its position is vulnerable due to an over-reliance on low-value-added assembly. The main findings of this research underscore that the sector’s long-term sustainability depends not only on geographical advantages or tax incentives such as the IMMEX program but also on a deliberate transition toward more-complex activities within the value chain, such as R&D and product design. Through a literature review and the development of a problem taxonomy, it was identified that the implementation of disruptive technologies, specifically additive manufacturing and the IoT, combined with circular economy models, has the potential to significantly reduce environmental impact and improve resilience to supply chain disruptions. Additionally, hybrid MCDM methods, such as AHP and TOPSIS, were presented as essential technical tools for eliminating subjectivity in supplier selection and prioritizing critical success factors.

Despite its contributions, the study acknowledges certain limitations. The current methodology relies heavily on synthesizing the existing literature and theoretical frameworks. While these frameworks are robust, they require more-extensive empirical validation using real-time data from the Mexican industry. Additionally, the study is dependent on expert opinions, which could introduce geographical or disciplinary biases into the results if not managed through fuzzy logic or neutrosophic methods.

In future work, the application of the FUCOM methodology is proposed to compare its consistency with traditional MCDM methods. Additionally, it is crucial to develop case studies applying the proposed innovation management flow to specific devices under COFEPRIS and FDA regulations. This will validate TRLs, MRLs, CRLs, and PRLs in real-world scenarios.

It is important to note that, although this research used the Baja California State ecosystem in Mexico as a reference point due to its industrial density, the proposed management model, taxonomy, and algorithms are scalable and transferable. Thus, this framework can easily be applied to other regions or countries with similar manufacturing contexts, such as Costa Rica, as well as to developing countries with growing technology clusters that are seeking to transition from a manufacturing model to a knowledge-based economy that focuses on sustainable innovation.

## Figures and Tables

**Table 1:** Comparative analysis of the most relevant research papers.

Element of Taxonomy	Publication	Technical Contribution	Methodology	Performance Metrics	Limitations and Restrictions
2.1.1, 2.3.1, 2.3.3	Nejad (2022) [8]	Prioritizing critical sustainability, Industry 4.0, and *leagile* (lean and agile) indicators in supplier selection.	Review of the previous literature and use of RBWM. Comparison using the AHP-TOPSIS method.	Determining sustainability and agility as critical factors for selecting suppliers.	The analysis overlooks the customer factor and fails to account for material-specific performance, suggesting that future research should incorporate the QFD and recognize that a supplier’s suitability may vary depending on the specific supply being sourced.
2.1.2, 2.2.1, 2.2.4	Bai et al., (2024) [7]	Evaluation of different scenarios to promote sustainability through manufacturing and remanufacturing. The impact of government subsidies is also assessed.	Comparison of three game models using mathematical analysis based on the previous literature and the law of diminishing returns.	Mathematical demonstration of higher performance in a system where the OEM certifies the remanufacturing company. Qualitative recommendations to consider remanufacturing as a viable alternative.	A strictly mathematical–theoretical methodology that does not consider factors such as business strategies, quality level, and regulatory frameworks.
2.2.2, 2.2.4, 2.3.1	Leong (2025) [10]	Development of a circular economy through additive manufacturing, reducing environmental impact, and taking into consideration applicable regulations.	Comparison of medical device archetypes, measuring the potential of the circular economy using TOPSIS.	A reduction of between 38% and 68% in greenhouse gas emissions, with energy savings of 54%. Qualitatively, a roadmap for Industry 5.0 is suggested, bringing the product closer to the user through additive manufacturing.	The range of applications of additive manufacturing and the properties of the materials used reduce the options of products that can be produced by this technology currently.
2.1.1	Momena et al. (2025) [13]	Definition of critical factors for the sustainability of supply chains based on quantitative/qualitative analysis, definition of arithmetic operations for sets of PLTs.	MCDM combined with PLTs is the solution to uncertainty in the system.	Identifying “Collaboration and Communication” as a critical factor for sustainability. Using the subscript degree and deviation degree functions.	The emphasis is firmly on the supply chain, with a carefully selected group of contributors. The study is biased, as most of the assessments and conclusions are derived from the opinions of two experts. The study also suggests future analyses that consider fuzzy numbers.
2.1.1, 2.2.2, 2.3.2	Sathiya (2023) [11]	Integration of the IoT and blockchain to strengthen supply chains in the medical supply industry.	Use of IoT, blockchain, and analytics through DEMATEL.	Proposed chain of things (CoT) model to strengthen the supply chain.	DEMATEL may present problems when encountering uncertain or incompatible information; therefore, the use of neutrosophic logic is proposed.
2.1.1, 2.3.2	Agrawal et al. (2025) [15]	Identifying critical factors in supply chains under a circular logistics system.	Fuzzy DEMATE	Identifying “long-term planning” as the main factor. Identifying “corporate social responsibility” as the main enabler.	Although supported by the DEMATEL methodology, it utilizes the knowledge of Indian experts, which may bias the study and influence the results within the specific context of India. Furthermore, the research findings suggest further investigation using fuzzy AHP, fuzzy TOPSIS, and fuzzy FUCOM.
2.4.1, 2.4.3	Gereffi & Hamrick (2026) [4]	Industrial upgrade to the medical device and aerospace industries, specifically in Baja California, Mexico.	Comprehensive analysis of the medical device manufacturing sector in Baja California, background review.	Recommendations for public policy in Baja California that encourage the development of both clusters in the region.	Conclusions based qualitatively on historical information, without a quantitative basis.
2.2.3	Santa et al. (2022) [9]	Determining the effect of human capital on the performance of companies of different sizes.	Hypothesis confirmation using equation models, based on company surveys.	It is concluded that human capital has an indirect positive impact on organizational performance, in combination with cost and quality policies.	The study focused on the agro-industrial region of Colombia.

## Data Availability

No new data were created or analyzed in this study. Data sharing is not applicable to this article.

## Abbreviations

The following abbreviations are used in this manuscript:

AHP	Analytic Hierarchy Process
CMs	Contract Manufacturers
CoCoSo	Combined Compromise Solution
COPRAS	Complex Proportional Assessment
CRLs	Customer Readiness Levels
DEMATEL	Decision-Making Trial and Evaluation Laboratory
IoT	Internet of Things
MCDM	Multi-Criteria Decision Making
MRLs	Manufacturing Readiness Levels
OEM	Original Equipment Manufacturing
PRLs	Policy Readiness Levels
R&D	Research and Development
TOPSIS	Technique for Order of Preference by Similarity to Ideal Solution
TRLs	Technology Readiness Levels
WASPAS	Weighted Aggregate Sum of Products Assessment
IMMEX	Industria Manufacturera, Maquiladora y de Servicios de Exportación
COFEPRIS	Comisión Federal para la Protección contra Riesgos Sanitarios
QFD	Quality Function Deployment
RBWM	Robust Best–Worst Method
PLTs	Probabilistic Language Terms
FUCOM	Full Consistency Method
FDA	Food and Drug Administration
EMA	European Medicines Agency
